# Supplementary material for: Optimization of differentiation and transcriptomic profile of THP-1 cells into macrophage by PMA
Source: PLoS One. 2023 Jul 17;18(7):e0286056. doi: 10.1371/journal.pone.0286056 (PMC10351730; doi:10.1371/journal.pone.0286056)
Supplement: S1 File — (ZIP) [file pone.0286056.s001.zip › supporting files/RNA-seq report.zip!/RNA-seq report/2_GeneQuantification_result/PCA_plot_2D.pdf]

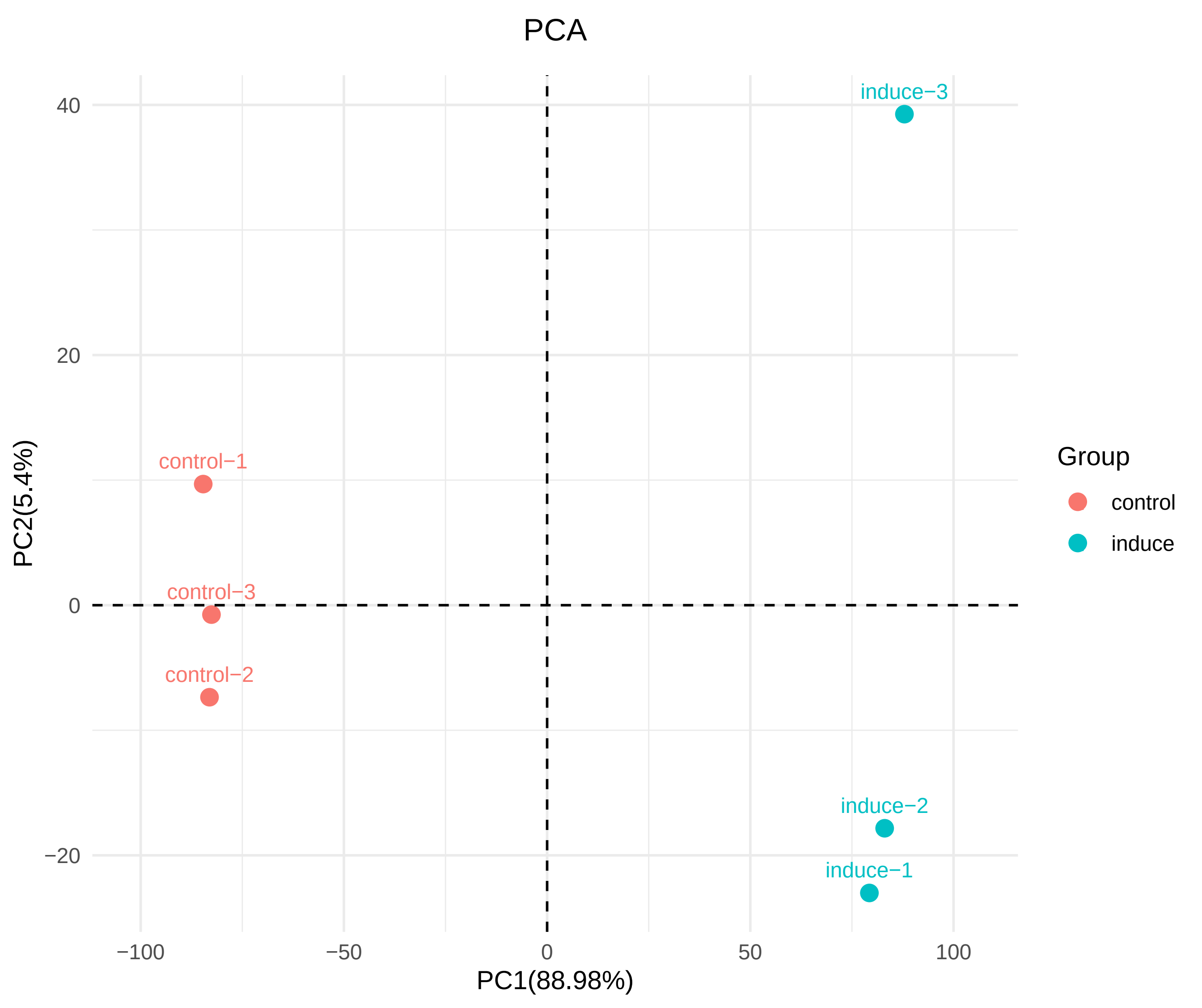
PCA
induce−3
control−1
control−3
control−2
induce−2
induce−1
40
20
0
−20
−100
−50
0
50
100
PC1(88.98%)
PC2(5.4%)
Group
control
induce
